# A Synergistic Remote Sensing Inversion Study of Water Depth in Inland Lakes Integrating Chlorophyll-a Concentration and Optical Indices

**DOI:** 10.3390/s26123780

**Published:** 2026-06-13

**Authors:** Junzhen Meng, Yunfei Wang, Jiajun Ren, Liya Xu, Linnan Fan

**Affiliations:** School of Geomatics and Geographical Information Engineering, North China University of Water Resources and Electric Power, Zhengzhou 450045, China; wangyunfei202303@163.com (Y.W.); 15534352091@163.com (J.R.); bawueryi@163.com (L.X.); 15209215760@163.com (L.F.)

**Keywords:** bathymetric retrieval, chlorophyll-a, inland lakes, machine learning

## Abstract

Accurate bathymetric information for inland lakes is essential for water resource management, ecological monitoring, and environmental research. However, the accuracy and robustness of remote sensing-based bathymetric retrieval are often constrained by the complex optical properties of inland waters and the limited representation of conventional inversion features. To address these challenges, this study systematically compared the performance of a multiband logarithmic ratio model and three machine learning models, including Random Forest (RF), XGBoost, and AdaBoost, for inland lake bathymetric retrieval. Furthermore, a synergistic retrieval framework integrating chlorophyll-a concentration (Chla) and a Water Optical Index (WOI) was proposed. The results show that: (1) The overall accuracy of the Random Forest, XGBoost, and AdaBoost models constructed with the integration of chlorophyll-a concentration and WOI ($R^{2}=0.93$, 0.93, and 0.91; MAE =0.06 m, 0.07 m, and 0.12 m; RMSE =0.14 m, 0.14 m, and 0.16 m) outperforms that of models using only multispectral band information ($R^{2}=0.93$, 0.91, and 0.82; MAE =0.06 m, 0.07 m, and 0.14 m; RMSE =0.14 m, 0.16 m, and 0.22 m). Moreover, all these machine learning models significantly outperform the traditional numerical model ($R^{2}=0.27$; MAE =0.29 m; RMSE =0.45 m), with the Random Forest model achieving the best overall performance. This indicates that the proposed method offers higher applicability and retrieval accuracy in complex inland lake environments. (2) The optimal Random Forest model integrating chlorophyll-a concentration and WOI achieved high-precision bathymetric inversion for inland lakes ($R^{2}=0.93$, MAE =0.06 m, RMSE =0.14 m). Based on the three-dimensional bathymetry derived from this model, the estimated lake storage capacity was $1072.11\times 10^{4}$ m^3^, compared with a measured volume of $1094.27\times 10^{4}$ m^3^, yielding a relative error of −2.03%. This result provides reliable and highly accurate data to support water resource management.

## 1. Introduction

As a key component of surface water resources, lakes play an irreplaceable role in maintaining ecological balance and supporting socio-economic development [1,2]. Accurate bathymetric information is essential for understanding hydrological processes, estimating water storage capacity, and supporting water resource management and environmental monitoring [3]. Although traditional bathymetric surveying techniques can provide highly accurate measurements, their application is constrained by vessel draft limitations, intensive field operations, high labor costs, and low surveying efficiency, making large-scale bathymetric mapping difficult [4,5]. In contrast, remote sensing technology offers an efficient alternative owing to its synoptic observation capability, wide spatial coverage, and high temporal resolution [6,7,8]. Consequently, remote sensing-based bathymetric retrieval has become an active research area, and three major methodological categories have gradually emerged.

Theoretical analytical models establish quantitative relationships between radiance and water depth by solving radiative transfer equations. Their main advantage lies in their strong physical basis; however, they require accurate estimation of inherent optical properties, such as absorption and scattering coefficients, which exhibit considerable spatial and temporal variability and are often difficult to obtain in practice [9]. Semi-theoretical and semi-empirical models combine radiative transfer theory with empirical regression approaches to establish relationships between remote sensing features and water depth. These models provide a balance between physical interpretability and operational simplicity; however, their dependence on predefined functional relationships may limit their performance in optically complex inland waters characterized by eutrophication and heterogeneous bottom substrates [10,11]. Statistical and machine learning models can directly learn nonlinear relationships between spectral information and water depth from observational data [12,13]. Recent studies have demonstrated that machine learning algorithms, such as Random Forest and XGBoost, can effectively capture complex spectral–bathymetric relationships, resulting in improved retrieval accuracy and robustness compared with conventional approaches [14,15].

Current developments in bathymetric retrieval have evolved from the use of raw spectral bands to the integration of derived optical features and, more recently, to the incorporation of water-quality parameters. However, systematic investigations of this progression remain limited. Most existing studies focus primarily on comparing algorithm performance using fixed input variables, while insufficient attention has been paid to the construction and evaluation of feature sets that explicitly reflect the optical attenuation processes governing water-depth retrieval [16,17,18,19]. Chlorophyll-a (Chl-a), a key indicator of phytoplankton biomass, directly influences the absorption and scattering characteristics of water bodies and therefore affects the propagation of light within the water column. Ignoring its spatiotemporal variability may introduce substantial uncertainty into bathymetric retrieval, particularly in optically complex and eutrophic lakes. Nevertheless, studies that systematically integrate Chl-a with multispectral bands and derived optical features within a unified bathymetric retrieval framework remain scarce, and the contribution of such water-quality information to retrieval performance has not been fully quantified [20,21]. To address this gap, Yuehai Lake, located in the Yinchuan Plain of northwestern China, was selected as the study area. High-accuracy Chl-a retrieval models and multiple bathymetric retrieval models were developed, and Chl-a and Water Optical Indices (WOIs) were introduced as synergistic variables. Their contributions to bathymetric retrieval accuracy were systematically evaluated, providing a practical framework for high-precision bathymetric mapping in inland lakes with complex optical characteristics.

## 2. Data and Preprocessing

### 2.1. Study Area

**Figure 1:**
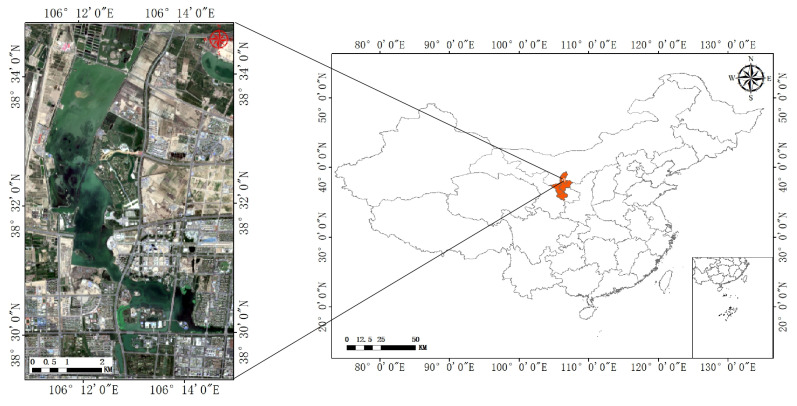
Schematic diagram of the study area.

As shown in Figure 1, the study area is located in Jinfeng District, Yinchuan City, Ningxia Hui Autonomous Region, China. Its geographic coordinates range from $106^{\circ }19^{\prime }$ to $106^{\circ }30^{\prime }$ E and from $38^{\circ }50^{\prime }$ to $38^{\circ }59^{\prime }$ N. The terrain generally slopes from west to east, with elevations ranging from approximately 1106 to 1113 m above sea level. The total area of the lake region is 16.44 km^2^, including approximately 8.00 km^2^ of open water with an average depth of 2.5–3.0 m and 4.95 km^2^ of marsh wetlands. The lakes and wetlands are closely interconnected, forming a continuous aquatic–terrestrial transition zone. Geomorphologically, the region is characterized by fluvial–lacustrine plains and is mainly composed of two representative landscape units: eolian landforms and alluvial depressions developed at the toes of alluvial fans. The lakebed is primarily composed of fine-grained sediments interspersed with aquatic vegetation, while water depth exhibits relatively gradual spatial variations. These characteristics make the area a representative site for studies of lake ecology, aquatic environments, and wetland conservation. Owing to its location within the urban area of Yinchuan and its close connection to the Yellow River irrigation system, the lake is primarily replenished by water diverted from the Yellow River.

### 2.2. Bathymetric Data

In-situ water depth data were collected between 21 and 28 June 2022 using an unmanned survey vessel equipped with a single-beam echo sounder and a real-time kinematic (RTK) GPS system. The horizontal and vertical positioning accuracies were 3 mm and 6 mm, respectively. The acquired measurements were processed to generate a high-precision bathymetric point cloud, which was subsequently interpolated and converted into 30 m spatial resolution raster data corresponding to the Landsat-9 pixel grid for model development and validation.

**Figure 2:**
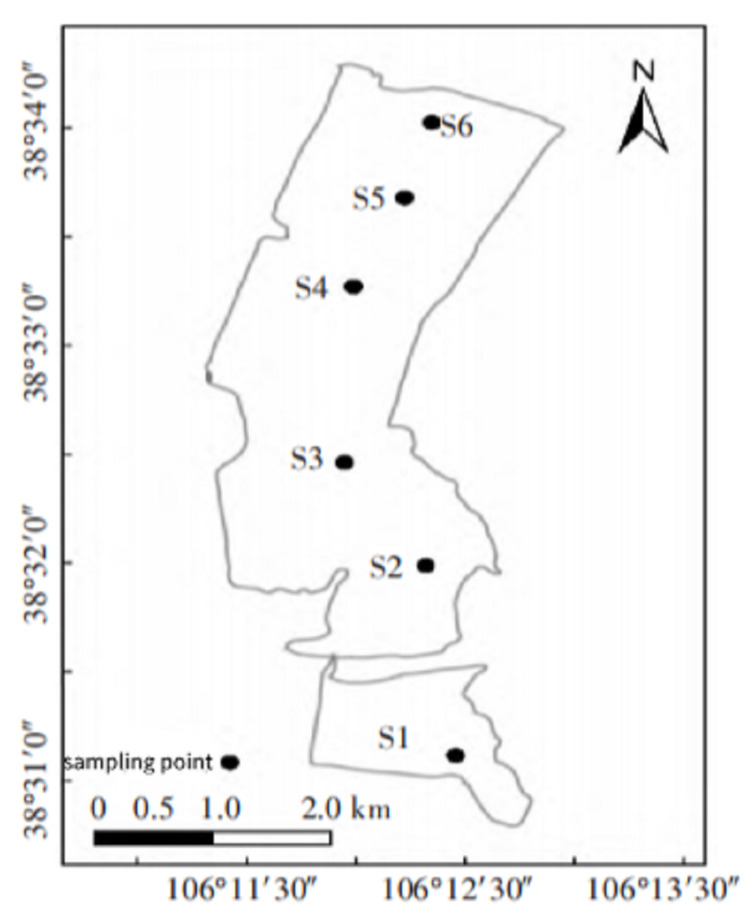
Distribution of Water Sample Collection Sites at Yuehai Lake.

Water samples were collected from six representative sampling sites (S1–S6; Figure 2) in Yuehai Lake during January, April, July, and October of 2020 and 2022. Chlorophyll-a (Chl-a) concentrations were determined in the laboratory using a fluorescence-based HYDROLAB Chl-a analyzer. These measurements were used as reference data for the calibration and validation of the Chl-a retrieval model.

### 2.3. Satellite Imagery Data

Water depth inversion image: A Landsat-9 Level-2 surface reflectance image acquired on 5 April 2022 (path/row: 129/34) was used for bathymetric retrieval. This product provides geometrically corrected surface reflectance data suitable for quantitative remote sensing applications. The image was acquired under clear-sky conditions with stable atmospheric characteristics over the study area, ensuring high-quality input data for water depth inversion. In addition, meteorological records from Yinchuan between 5 April and 29 June 2022 indicated the absence of extreme precipitation or drought events that could substantially alter lake water levels. Moreover, no significant anthropogenic disturbances were reported in the Yuehai Lake area during this period. Therefore, the Landsat-9 image acquired on 5 April 2022 was considered representative of the lake conditions and was selected for bathymetric retrieval.

Chl-a inversion imagery: A total of nine Sentinel-2 multispectral images were used to retrieve chlorophyll-a (Chl-a) concentration, covering January, April, July, and October of 2020 and 2021, together with one image acquired in June 2022. All images were obtained and preprocessed on the Google Earth Engine platform, including radiometric calibration, atmospheric correction, and cloud masking.

## 3. Bathymetric Estimation Modeling

### 3.1. Bathymetric Estimation Factor Selection

Water depth retrieval is influenced by both the optical characteristics of the water body and the selection of inversion variables. Different spectral features therefore exhibit varying sensitivities to water depth. Pearson correlation analysis conducted for Yuehai Lake showed that the relationships between individual spectral bands and measured water depth were generally weak to moderate. Specifically, the correlation coefficients for bands B1–B3 were approximately 0.39, whereas the coefficient decreased to 0.37 for B4 and further declined to 0.20–0.25 for B5–B7. This progressive reduction in correlation suggests that the sensitivity of spectral reflectance to water depth gradually weakens with increasing wavelength, indicating that individual bands alone are insufficient to adequately characterize water depth variability.

Further analysis of the Water Optical Indices (WOIs) revealed substantial differences in their relationships with water depth (Table 1). The traditional water indices NDWI and MNDWI exhibited weak correlations (r≈0.08–0.14), suggesting limited sensitivity to bathymetric variations. In contrast, WOI1 and WOI2 showed relatively stronger correlations (r≈0.39–0.40), while WOI4 exhibited a moderate correlation (r=0.15). The remaining indices displayed weak or negative correlations, with WOI5 showing the strongest negative relationship (r=−0.33). These results indicate that different optical indices capture distinct aspects of the underwater optical environment and vary considerably in their ability to represent water depth. Overall, the relationships between individual inversion variables and water depth were relatively weak, highlighting the limitations of single-feature approaches. Therefore, integrating multiple optical features and water-quality-related variables, together with nonlinear modeling techniques, may provide a more effective strategy for improving bathymetric retrieval accuracy in optically complex inland waters.

### 3.2. Methodology

#### 3.2.1. Methods for Estimating Chlorophyll a Concentrations and Synergistic Inversion

In remote sensing-based bathymetric retrieval, the optical properties of the water column play a critical role in determining retrieval accuracy. As one of the primary optically active constituents in inland waters, chlorophyll-a (Chl-a) influences the propagation of light within the water column by modifying absorption and scattering processes. Variations in Chl-a concentration can therefore affect the spectral response of water bodies and introduce uncertainty into bathymetric retrieval. To account for these effects, a synergistic retrieval framework integrating Chl-a concentration and Water Optical Indices (WOIs) was developed. Following the preprocessing of Sentinel-2 imagery, a series of empirical models, including single-band, band-difference, and logarithmic-ratio models, were constructed and evaluated. The optimal model formulations were selected based on the performance metrics $R^{2}$, RMSE, and MAE. The resulting optimal model equations are presented as follows:

(1) $$\mathrm{Chl}-a1=6320.19{\left(\frac{\ln(B3)}{\ln(B10)}\right)}^{2}-2856.89\left(\frac{\ln(B3)}{\ln(B10)}\right)+324.47$$

(2) $$\mathrm{Chl}-a2=6611.95{\left(\frac{\ln(B2)}{\ln(B10)}\right)}^{2}-3017.96\left(\frac{\ln(B2)}{\ln(B10)}\right)+337.04$$

In the equations, B2, B3, and B10 denote the blue, green, and short-wave infrared (SWIR) bands, respectively.

A Random Forest (RF) model was developed in Python using multispectral reflectance as input features, with the dataset randomly divided into training and validation subsets at a ratio of 7:3. Among all evaluated models, the RF model achieved the best performance for Chl-a retrieval, with $R^{2}=0.80$, RMSE=14.25μg/L, and MAE=12.42μg/L, outperforming the empirical models. The retrieved Chl-a concentration data were subsequently spatially and temporally matched with Landsat-9 imagery through image registration and resampling. The resulting Chl-a information was then incorporated into the bathymetric retrieval framework as a synergistic variable. To evaluate the contribution of different feature combinations, four input schemes were designed: Band, Band + WOI, Band + Chl-a, and Band + WOI + Chl-a. This strategy enabled a systematic assessment of the transition from single-source spectral information to the integration of multisource optical and water-quality-related features.

#### 3.2.2. Bathymetric Estimation Model

(1) Multi-Band Logarithmic Ratio Model

The MLR model is an extension of the two-band model, suitable for variations in water types within a single category. Even with sufficient spectral data, it can also handle scenarios where both water and bottom types change within a single scene [22]. The formula is as follows:

(3) $$\begin{array}{cc} Z & =m_{0}+m_{1}\times \frac{\ln\left[n\times R_{w}\left(\lambda_{2}\right)\right]}{\ln\left[n\times R_{w}\left(\lambda_{1}\right)\right]}+\frac{\ln\left[n\times R_{w}\left(\lambda_{3}\right)\right]}{\ln\left[n\times R_{w}\left(\lambda_{2}\right)\right]}+\cdots +\frac{\ln\left[n\times R_{w}\left(\lambda_{n}\right)\right]}{\ln\left[n\times R_{w}\left(\lambda_{n-1}\right)\right]} \end{array}$$

(2) Machine Learning Model

Machine Learning Model Based on the Python platform, three machine learning models for bathymetric retrieval were developed, including Random Forest (RF) [23], AdaBoost [24], and XGBoost [25]. The dataset consisted of 9154 training samples and 3921 testing samples. The hyperparameter configurations of the three models for Yuehai Lake are summarized in Table 2, Table 3 and Table 4.

### 3.3. Precision Evaluation

This study uses three metrics, mean absolute error (MAE), root mean square error (RMSE), and coefficient of determination (R^2^), to evaluate the performance of the model. The formulas are as follows:

(4) $$R^{2}=1-\frac{\sum_{i=1}^{n}{\left(y_{i}-{\hat{y}}_{i}\right)}^{2}}{\sum_{i=1}^{n}{\left(y_{i}-{\bar{y}}_{i}\right)}^{2}}$$

(5) $$RMSE=\sqrt{\frac{\sum_{i=1}^{n}{\left(y_{i}-{\hat{y}}_{i}\right)}^{2}}{n}}$$

(6) $$MAE=\frac{\sum_{i=1}^{n}\left|y_{i}-{\hat{y}}_{i}\right|}{n}$$

where $y_{i}$ represents the measured depth, ${\hat{y}}_{i}$ describes the estimated depth, $\bar{y_{i}}$ represents the mean value of the measured depths, and n indicates the number of input data points.

## 4. Results

### 4.1. Optimization Analysis of Water Depth Models Using Chlorophyll a and Water Optical Indices

**Figure 3:**
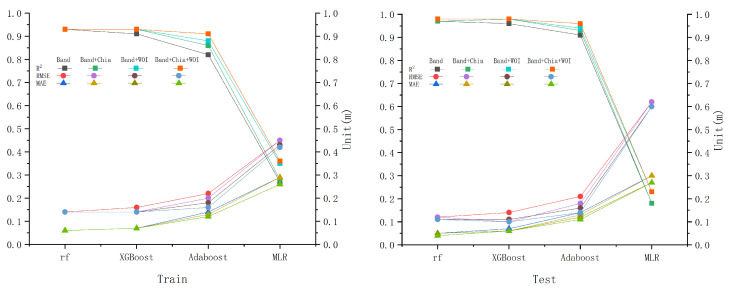
Comparative Analysis of Water Depth Inversion Accuracy for Various Models of Yuehai Lake.

This study further evaluated the effects of Chl-a concentration and Water Optical Indices (WOIs) on bathymetric retrieval performance. As shown in Table 5 and Figure 3, the traditional multiband logarithmic ratio model exhibited relatively low accuracy across all input combinations, with test-set $R^{2}$ values ranging from 0.27 to 0.36 and RMSE values ranging from 0.42 to 0.45 m. In contrast, the machine learning models showed greater responsiveness to the incorporation of Chl-a concentration and WOIs. Among them, the AdaBoost model exhibited the most pronounced improvement, with the test-set $R^{2}$ increasing from 0.82 to 0.91 and the RMSE decreasing from 0.22 m to 0.16 m. The XGBoost model also benefited from the additional features, maintaining a high level of accuracy with $R^{2}=0.93$ and RMSE=0.14 m. The Random Forest model achieved the best overall performance and robustness, consistently yielding a test-set $R^{2}$ of 0.93 and an RMSE of 0.14 m. Overall, the integration of Chl-a concentration and WOIs contributed to improved retrieval accuracy and enhanced the ability of the models to characterize optically complex inland waters, demonstrating the value of combining water-quality information with optical features for bathymetric retrieval.

**Figure 4:**
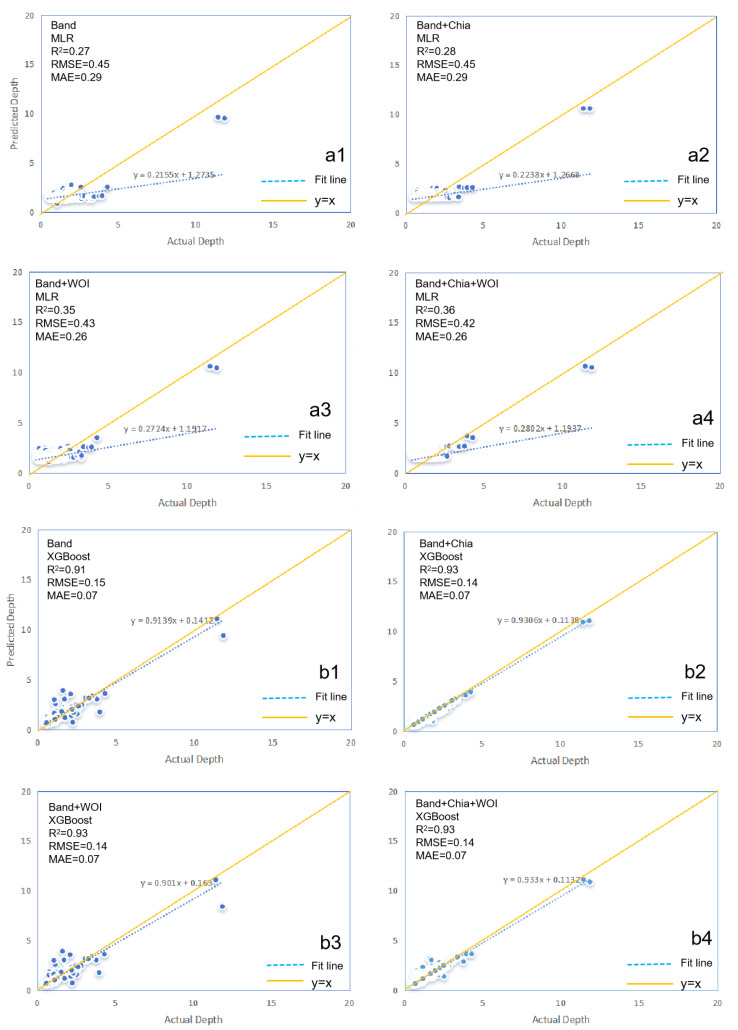

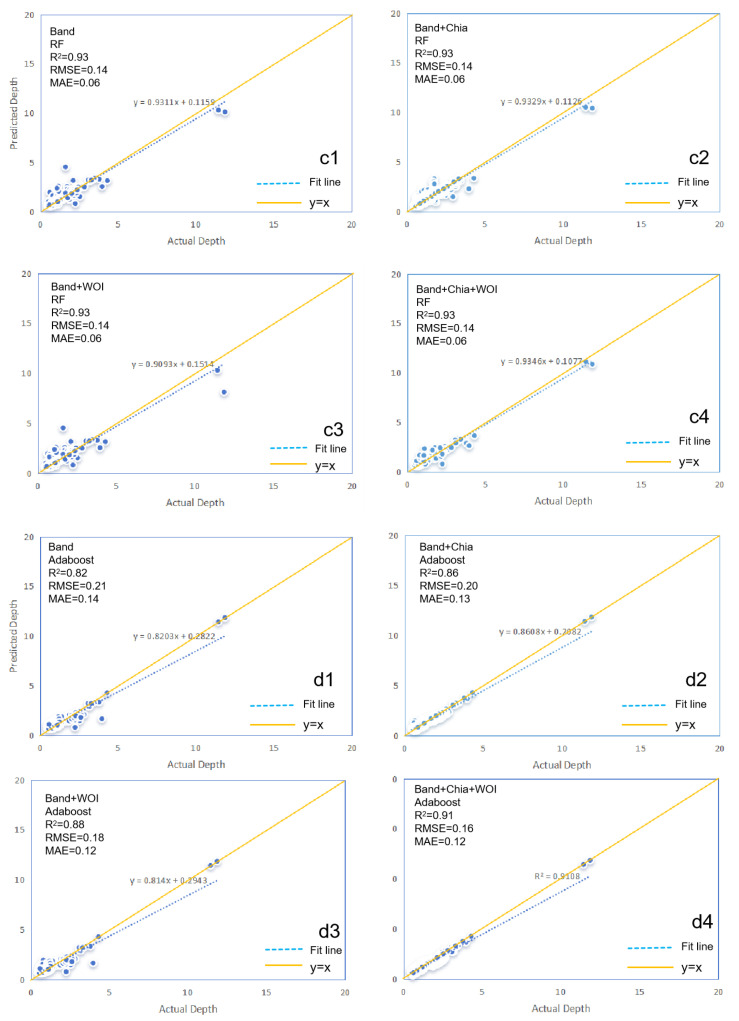
Scatter plot (**a1**–**d4**).

To further evaluate the effects of feature optimization on model performance, scatter plots comparing predicted and observed water depths for the test set were generated for each model (Figure 4). Figure 4(a1–a4) correspond to the multiband logarithmic ratio model using the Band, Band + Chl-a, Band + WOI, and Band + Chl-a + WOI input schemes, respectively. The same arrangement is adopted for the AdaBoost and XGBoost models shown in panels (b) and (c).

As shown in Figure 4, the multiband logarithmic ratio model exhibited relatively poor performance under all input combinations, with test-set $R^{2}$ values ranging from 0.27 to 0.36. The fitted regression lines deviated substantially from the y=x line, indicating limited capability to capture the nonlinear relationships between spectral information and water depth in optically complex inland waters. In contrast, the machine learning models achieved considerably higher accuracy and showed different responses to feature enhancement. Using only spectral bands as inputs, AdaBoost achieved a test-set $R^{2}$ of 0.82, although noticeable deviations from the y=x line remained, particularly in shallow-water areas. The incorporation of Chl-a increased the test-set $R^{2}$ to 0.86 and reduced the discrepancy between predicted and observed values. Further inclusion of WOIs improved the test-set $R^{2}$ to 0.91 and reduced the RMSE to 0.16 m, suggesting that Chl-a and WOIs effectively enhance the model’s ability to characterize water-depth variability across different depth ranges. XGBoost achieved high accuracy even when using only spectral bands ($R^{2}=0.91$). After incorporating Chl-a and WOIs, the model maintained a test-set $R^{2}$ of 0.93 while exhibiting a more concentrated distribution of prediction points, particularly in deeper-water regions, indicating improved representation of bathymetric variability. Among all models, Random Forest demonstrated the highest stability, consistently maintaining a test-set $R^{2}$ of 0.93 across all input combinations. The inclusion of Chl-a and WOIs further reduced prediction errors, improved the agreement between the fitted regression line and the y=x line, and resulted in a more uniform distribution of predictions within the 0–5 m depth range.

Overall, all three machine learning models exhibited systematic prediction bias in shallow-water areas, characterized by underestimation at greater depths and slight overestimation in very shallow regions. The incorporation of Chl-a and WOIs effectively reduced these biases and improved model robustness. In terms of overall performance, XGBoost achieved the highest predictive accuracy, particularly in deeper-water areas, whereas Random Forest exhibited the greatest stability across different feature combinations. These results highlight the advantages of integrating water-quality and optical features into machine learning frameworks and demonstrate the suitability of Random Forest and XGBoost for bathymetric retrieval in optically complex inland lakes.

### 4.2. Bathymetric Inversion Results of Yuehai Lake

**Figure 5:**
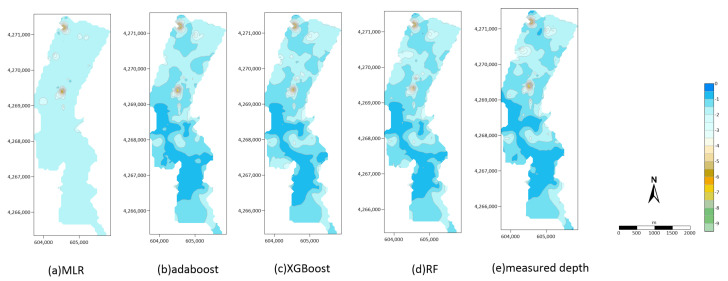
Map of water depth inversion results for Yuehai Lake.

A 2D bathymetric map of Yuehai Lake with a spatial resolution of 30 m was generated using the Band+WOI+Chl-a retrieval framework (Figure 5) and visualized using a gradient color scheme. Water depth was classified into eight continuous intervals with 0.5 m increments, ranging from light blue (0–1 m) to dark blue (>5 m), while contour lines at 0.5 m intervals were superimposed to enhance the representation of underwater topographic features. The resulting bathymetric map reveals a distinct spatial pattern characterized by deeper central regions and relatively shallow northern and southern zones. The central deep-water area (water depth > 5 m) is represented by dense, closed contour lines, indicating pronounced depth gradients, whereas the northern shallow-water region (water depth < 2 m) occupies approximately 40% of the lake area and exhibits relatively uniform depth distribution with widely spaced contours, reflecting a gently varying underwater terrain.

At the local scale, the bathymetric map successfully captures several representative geomorphological features, including multiple isolated deep depressions in the central lake area, a ridge-like topographic structure extending in a northwest–southeast direction in the northern region, and several small depressions distributed throughout the eastern section of the lake. These features demonstrate the ability of the proposed retrieval framework to characterize spatial heterogeneity in underwater topography. Furthermore, the spatial distribution of water depth exhibits a clear correspondence with the retrieved Chl-a patterns. Areas with greater water depth generally coincide with relatively low Chl-a concentrations, whereas shallower regions tend to correspond to comparatively higher Chl-a concentrations. This spatial consistency suggests that incorporating Chl-a information contributes to improved characterization of optically complex water environments and supports the effectiveness of the synergistic retrieval framework.

Although a certain degree of terrain smoothing remains evident in areas with abrupt depth variations, and uncertainties associated with aquatic vegetation may affect retrieval accuracy in some shallow-water regions, the overall bathymetric map shows good agreement with field measurements. Combined with the achieved retrieval accuracy (RMSE=0.14 m), the results demonstrate that the proposed framework can provide reliable bathymetric information for lake hydrological analysis, underwater terrain characterization, and water resource assessment.

## 5. Discussion

### 5.1. Analysis of the Three-Dimensional Topographic Reconstruction Capabilities of Water Depth Inversion

**Figure 6:**
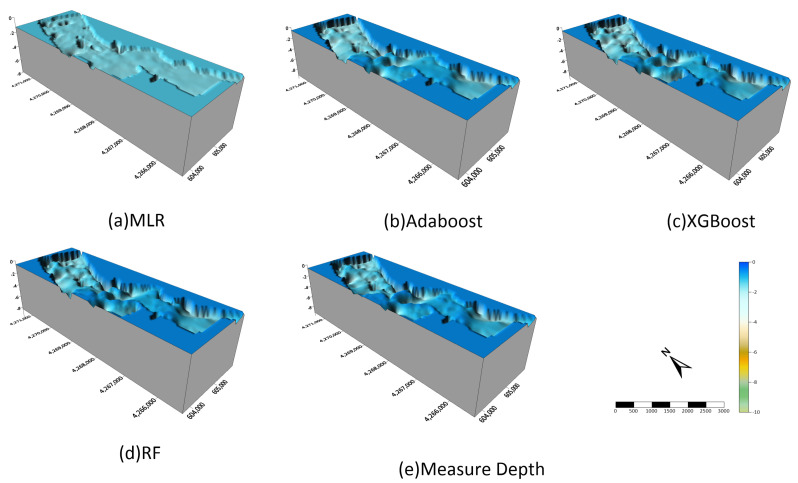
Three-dimensional topographic distribution map under Yuehai Lake (elevation in m, coordinates in m).

In terms of topographic representation, three-dimensional visualization provides a more comprehensive characterization of underwater terrain than conventional two-dimensional bathymetric maps. As shown in Figure 6, the reconstructed three-dimensional bathymetry clearly depicts the spatial structural characteristics of Yuehai Lake. The transition between deep- and shallow-water regions is readily identifiable, with the central deep-water area exhibiting a pronounced concave morphology and relatively steep depth gradients, while the northern region is characterized by gently sloping terrain with localized undulations. The three-dimensional surface further reveals fine-scale geomorphological features, including the sidewall morphology and bottom relief of isolated deep depressions, as well as ridge-like structures and small depressions distributed within shallow-water areas. These features are represented with strong spatial continuity and provide an intuitive visualization of underwater terrain variability.

Compared with two-dimensional bathymetric maps, which primarily rely on color gradients and contour patterns to represent depth variations, the three-dimensional model simultaneously displays bathymetric magnitude and geomorphological structure, thereby improving the visualization of local terrain features and depth gradients. The reconstructed surfaces generated by different models exhibit distinct characteristics. The Random Forest model produced a spatially continuous bathymetric surface with well-defined topographic features, whereas the XGBoost model showed a tendency toward localized smoothing in some shallow-water regions. The AdaBoost model captured the major terrain patterns but exhibited greater surface fluctuations, while the multiband logarithmic ratio model displayed reduced spatial coherence and weaker representation of fine-scale topographic features. These differences indicate that machine learning models are generally more effective than traditional empirical approaches in representing complex underwater terrain.

Overall, the three-dimensional bathymetric reconstruction provides a more intuitive and detailed depiction of underwater topography than two-dimensional representations. The results demonstrate that the proposed retrieval framework can effectively support underwater terrain characterization and offers valuable information for lake geomorphological analysis, hydrological investigations, and water resource assessment under optically complex conditions.

### 5.2. Analysis of the Accuracy of Reservoir Capacity Estimates

Compared with conventional two-dimensional bathymetric representations, lake storage-capacity estimation based on three-dimensional bathymetric reconstruction can better characterize the spatial variability and morphological complexity of underwater terrain, thereby providing a more robust basis for storage-capacity assessment. Using the three-dimensional bathymetric surfaces derived from the optimal retrieval models, lake storage capacity was estimated and compared with the measured value of Yuehai Lake ($1094.27\times 10^{4}$ m^3^). Among all models, the Random Forest (RF) model produced the most accurate estimate, yielding a storage capacity of $1072.11\times 10^{4}$ m^3^ and a relative error of only −2.03%. The XGBoost model achieved a comparable result, with an estimated storage capacity of $1071.75\times 10^{4}$ m^3^ and a relative error of −2.07%. The AdaBoost model estimated a storage capacity of $1061.80\times 10^{4}$ m^3^, corresponding to a relative error of −2.99%. In contrast, the traditional multiband logarithmic ratio (MLR) model produced a substantially larger estimate of $1284.26\times 10^{4}$ m^3^, resulting in a relative error of +17.37%, which was considerably higher than those obtained by the machine learning models (Table 6).

The ranking of storage-capacity estimation errors was generally consistent with the bathymetric retrieval performance of the corresponding models, suggesting that storage-capacity estimation can serve as an effective supplementary indicator for evaluating the reliability of bathymetric reconstruction results. Models with higher bathymetric retrieval accuracy tended to generate more accurate storage-capacity estimates, highlighting the close relationship between underwater terrain representation and volumetric assessment. In particular, the superior performance of the RF and XGBoost models indicates that machine learning approaches are more effective in capturing the complex spatial variability in optically complex inland waters.

Overall, storage-capacity estimation based on high-precision three-dimensional bathymetric reconstruction not only provides an independent validation of model performance but also offers valuable quantitative information for water resource assessment, hydrological analysis, ecological management, and lake monitoring applications.

## 6. Conclusions

This study systematically developed and evaluated a bathymetric retrieval framework for Yuehai Lake by integrating chlorophyll-a (Chl-a) concentration and Water Optical Indices (WOIs) into machine learning models. The main conclusions are as follows:

(1) The incorporation of Chl-a concentration and WOIs improved bathymetric retrieval performance to varying degrees across different models. The multiband logarithmic ratio model showed only limited improvement, with the test-set $R^{2}$ increasing from 0.27 to 0.36 and the RMSE decreasing to 0.42 m. In contrast, the machine learning models exhibited greater responsiveness to feature enhancement. The AdaBoost model showed the largest improvement, with the test-set $R^{2}$ increasing from 0.82 to 0.91 and the RMSE decreasing from 0.22 m to 0.16 m. The XGBoost model maintained consistently high performance, achieving a test-set $R^{2}$ of 0.93 and an RMSE of 0.14 m. Among all models, the Random Forest model achieved the best overall performance, with a test-set $R^{2}$ of 0.93, an RMSE of 0.14 m, and an MAE of 0.06 m. These results demonstrate that integrating water-quality information with optical features can effectively enhance bathymetric retrieval accuracy in optically complex inland lakes.

(2) Although the proposed framework achieved satisfactory retrieval accuracy in the study area, its transferability to other aquatic environments requires further investigation. Bathymetric retrieval performance may be influenced by factors such as water quality, depth range, bottom substrate characteristics, shoreline conditions, and atmospheric and environmental variability during image acquisition. In particular, elevated suspended sediment concentrations may increase water-column reflectance and lead to depth overestimation, whereas strong colored dissolved organic matter (CDOM) absorption may reduce spectral sensitivity to water depth. Future studies should incorporate additional water-quality and optical parameters, including suspended sediment concentration, CDOM, and sediment characteristics, and evaluate model performance across different lakes, seasons, and environmental conditions. Such efforts will contribute to a better understanding of the interactions between water-quality parameters and optical features and further improve the robustness and applicability of bathymetric retrieval models for inland waters.

## Figures and Tables

**Table 1:** Correlation Analysis of Inversion Factors and Measured Water Depths.

Estimation Factors		Estimation Factors	
Factor	Correlation (*r*)	Factor	Correlation (*r*)
B1	0.39	WOI3	0.08
B2	0.39	WOI4	0.39
B3	0.39	WOI5	0.40
B4	0.37	WOI6	0.10
B5	0.24	WOI7	0.15
B6	0.22	WOI8	−0.33
B7	0.20	WOI9	0.21
WOI1	0.14	WOI10	0.07
WOI2	0.14		

**Table 2:** AdaBoost model parameters.

Parameter	Value
n_estimators	350
loss	square
learning_rate	0.1
max_depth	8
min_samples_split	5
min_samples_leaf	5
max_features	‘sqrt’

**Table 3:** XGBoost model parameters.

Parameter	Value
max_depth	5
learning_rate	0.05
colsample_bytree	0.9
gamma	0.01
reg_alpha	0.1
subsample	0.85

**Table 4:** Random Forest model parameters.

Parameter	Value
max_depth	40
max_features	1
min_samples_leaf	1
min_samples_split	2
n_estimators	100

**Table 5:** Comparative Analysis of Water Depth Inversion Accuracy for Various Models of Yuehai Lake.

Model	Feature	R^2^		RMSE (m)		MAE (m)	
		Train	Test	Train	Test	Train	Test
MLR	Band	0.18	0.27	0.62	0.45	0.30	0.29
	Band + Chl-a	0.18	0.28	0.62	0.45	0.30	0.29
	Band + WOI	0.23	0.35	0.60	0.43	0.27	0.26
	Band + Chl-a + WOI	0.23	0.36	0.60	0.42	0.27	0.26
RF	Band	0.97	0.93	0.12	0.14	0.05	0.06
	Band + Chl-a	0.97	0.93	0.12	0.14	0.05	0.06
	Band + WOI	0.98	0.93	0.11	0.14	0.05	0.06
	Band + Chl-a + WOI	0.98	0.93	0.11	0.14	0.04	0.06
Adaboost	Band	0.91	0.82	0.21	0.22	0.14	0.14
	Band + Chl-a	0.93	0.86	0.18	0.20	0.13	0.13
	Band + WOI	0.94	0.88	0.16	0.18	0.12	0.12
	Band + Chl-a + WOI	0.96	0.91	0.14	0.16	0.11	0.12
XGBoost	Band	0.96	0.91	0.14	0.16	0.07	0.07
	Band + Chl-a	0.98	0.93	0.10	0.14	0.06	0.07
	Band + WOI	0.98	0.93	0.11	0.14	0.06	0.07
	Band + Chl-a + WOI	0.98	0.93	0.10	0.14	0.06	0.07

**Table 6:** Results for Different Models.

Model	Lake Storage ($10^{4}$ m^3^)
RF	1072.11
Adaboost	1061.80
XGBoost	1071.75
Measured storage	1094.27
MLR	1284.26

## Data Availability

The data presented in this study are available on request from the corresponding author.

## Abbreviations

The following abbreviations are used in this manuscript:

RF	Random forest
MLR	Multi-band logarithmic ratio

## References

[1] Zhang Z., Liu X. (2023). Bathymetric modelling for long-term monitoring of water dynamics of Ramsar-listed lakes using inundation frequency and photon-counting LiDAR data. Ecohydrol. Hydrobiol..

[2] Meng J., Yang X., Li Z., Zhao G., He P., Xuan Y., Wang Y. (2024). Tracking Evapotranspiration Patterns on the Yinchuan Plain with Multispectral Remote Sensing. Sustainability.

[3] Jia D., Li Y., He X., Yang Z., Wu Y., Wu T., Xu N. (2023). Methods to improve the accuracy and robustness of satellite-derived bathymetry through processing of optically deep waters. Remote Sens..

[4] Pydyn A., Popek M., Kubacka M., Janowski Ł. (2021). Exploration and reconstruction of a medieval harbour using hydroacoustics, 3-D shallow seismic and underwater photogrammetry: A case study from Puck, southern Baltic Sea. Archaeol. Prospect..

[5] Tang Y. (2023). Research on Shallow Water Depth Remote Sensing of Reefs Based on Machine Learning and Empirical Methods. Ph.D. Thesis.

[6] Cahalane C., Magee A., Monteys X., Casal G., Hanafin J., Harris P. (2019). A comparison of Landsat 8, RapidEye and Pleiades products for improving empirical predictions of satellite-derived bathymetry. Remote Sens. Environ..

[7] Lee Z., Shang S., Wang Y., Wei J., Ishizaka J. (2020). Nature of optical products inverted semianalytically from remote sensing reflectance of stratified waters. Limnol. Oceanogr..

[8] Duan X., Yi L., Li Q., Chen L., Zhang X. (2025). Bathymetric inversion of the Laguo Co Salt Lake, Xizang, based on multispectral images and machine learning. J. Salt Lake Res..

[9] Ma X. (2024). Research on Shallow Water Depth Inversion from Optical Remote Sensing Imagery. Ph.D. Thesis.

[10] Shah A., Deshmukh B., Sinha L. (2020). A review of approaches for water depth estimation with multispectral data. World Water Policy.

[11] Kutser T., Hedley J., Giardino C., Roelfsema C., Brando V.E. (2020). Remote sensing of shallow waters—A 50 year retrospective and future directions. Remote Sens. Environ..

[12] Sagawa T., Yamashita Y., Okumura T., Yamanokuchi T. Shallow Water Bathymetry Derived by Machine Learning and Multitemporal Satellite Images. Proceedings of the IGARSS 2019—2019 IEEE International Geoscience and Remote Sensing Symposium.

[13] Wen K., Li Y., Wang H. (2020). Bathymetry Retrieval Technology for Inland Waters Based on Remote Sensing and Machine Learning. Trop. Geogr..

[14] Shen W., Meng R., Luan K. (2023). Comparison of Four Machine Learning Models for Remote Sensing Bathymetry Inversion. Hydrogr. Surv. Charting.

[15] Meng J., Wang Y., Liu W., Yang X., He P. (2025). Research on the Development of an Inland Lake Bathymetry Estimation Model Based on Multispectral Data. Sensors.

[16] Duan Z., Zuo X., Yu K., Zheng X. (2023). An Optimized Coral Reef Bathymetry Inversion Method Based on ICESat-2 and GF-1 under Geomorphic Zonation. Sci. Geogr. Sin..

[17] Wang Z., Ma Z., Xiong Z. (2023). Evaluation of Shallow Water Depth Inversion Methods Using Multispectral Remote Sensing Data and Multiple Machine Learning Algorithms. Trop. Geogr..

[18] Zou W., Zhu G., Cai Y., Vilmi A., Xu H., Zhu M., Qin B. (2020). Relationships between nutrient, chlorophyll a and Secchi depth in lakes of the Chinese Eastern Plains ecoregion: Implications for eutrophication management. J. Environ. Manag..

[19] Cheruvelil K.S., Webster K.E., King K.B., Poisson A.C., Wagner T. (2022). Taking a macroscale perspective to improve understanding of shallow lake total phosphorus and chlorophyll a. Hydrobiologia.

[20] Zhang X., Li C., Liu C., Zhou W., Zhao J., He J., Xu Z., Yang Z., Cao W. (2025). Study on the underwater light field and water quality parameters along the coast around the Guangdong-Hong Kong-Macao Greater Bay Area. Opt. Express.

[21] Gao S., Shi Y., Zhang S., Gao C. (2024). Temporal and spatial variation patterns of chlorophyll a in marine ranching under global interannual events. Mar. Environ. Res..

[22] Lyzenga D.R., Malinas N.P., Tanis F.J. (2006). Multispectral bathymetry using a simple physically based algorithm. IEEE Trans. Geosci. Remote Sens..

[23] Han Z., Zhu X., Fang X., Wang Z., Wang L., Zhao G., Jiang Y. (2016). Hyperspectral Estimation of Apple Tree Canopy LAI Based on SVM and RF Regression. Spectrosc. Spectr. Anal..

[24] Richardson G., Foreman N., Knudby A., Wu Y., Lin Y. (2024). Global deep learning model for delineation of optically shallow and optically deep water in Sentinel-2 imagery. Remote Sens. Environ..

[25] Chen H., Wang Y., Liu G., Wu S., Chen H., Wu Z. (2025). Spectral-Geospatial XGBoost Regression for Bathymetric Mapping in Yellow River. Mar. Geod..

